# Epidemiology, prevalence, and associated factors of oral candidiasis in HIV patients from southwest Iran in post-highly active antiretroviral therapy era

**DOI:** 10.3389/fmicb.2022.983348

**Published:** 2022-09-02

**Authors:** Maryam Erfaninejad, Ali Zarei Mahmoudabadi, Elham Maraghi, Mohammad Hashemzadeh, Mahnaz Fatahinia

**Affiliations:** ^1^Department of Medical Mycology, School of Medicine, Ahvaz Jundishapur University of Medical Sciences, Ahvaz, Iran; ^2^Department of Medical Mycology, Infectious and Tropical Diseases Research Center, Health Research Institute, Ahvaz Jundishapur University of Medical Sciences, Ahvaz, Iran; ^3^Department of Biostatistics and Epidemiology, School of Health, Ahvaz Jundishapur University of Medical Sciences, Ahvaz, Iran; ^4^Department of Microbiology, School of Medicine, Ahvaz Jundishapur University of Medical Sciences, Ahvaz, Iran

**Keywords:** oral, candidiasis, HIV, AIDS, BMI, *Candida* species, Iran

## Abstract

**Background:**

Oral candidiasis (OC) is one of the most common opportunistic fungal infections among people living with HIV/AIDS (PLWHA). The prevalence of OC and *Candida* profiles among HIV-infected patients might be changing in the era of Highly Active Antiretroviral Therapy (HAART). This study aimed to identify *Candida* spp., determine OC prevalence and associated risk factors for PLWHA.

**Materials and methods:**

Oral candidiasis prevalence was explored in oral swabs of 276 patients who referred for consultation at Behavioral Diseases Counseling Center (BDCC). Clinical symptoms, culture and molecular assays were used for OC detection. In statistical analysis, we assessed socio-demographic characteristics, clinical information and treatment history of some infections.

**Results:**

The overall prevalence of OC was 41%. *Candida albicans* (64.6%) was the most common species, followed by *C. glabrata* (26.5%) and *C. dubliniensis* (19.5%). *Candida famata*, *C. africana*, and *C. stellatoidea* as the first fungi isolated from OC in PLWHA from southwest Iran. In 36.3% of patients, mixed cultures of more than one species were observed. Body mass index (BMI) (OR = 0.947; CI = 0.89–0.99; *p* = 0.045) and CD4 count ≤ 200 cells/mm^3^ (OR = 4.365; CI = 1.73–10.98; *p* = 0.002) were the predictors of OC in the final model of multiple logistic regression analysis. Education level, addiction status, sexual behaviors, chest X-ray, other infections and WHO clinical stage were other important risk factors for OC.

**Conclusion:**

Oral candidiasis remains a significant opportunistic infection in post-HAART era among PLWHA. Despite the increasing prevalence of NAC species, *C. albicans* (64.6%) was still the predominant species. Our results showed that low BMI with OC indicates treatment failure (i.e., failure to increase CD4 count or suppress viral load). Also, low CD4 counts (≤200 cells/mm^3^) in HIV patients show an impaired immune status, and our findings emphasize that OC can be a clinical indicator of HIV infection in individuals who do not know their HIV status or have failed treatment.

## Introduction

Oral candidiasis (OC) is the most common human fungal infection, which is known as a frequent opportunistic infection in immunosuppressed patients (Vila et al., 2020). Prolonged use of broad-spectrum antibiotics, treatment with corticosteroids, xerostomia, malignancies, Sjögren’s syndrome, acquired immune deficiency syndrome (AIDS), use of dentures and smoking are the predisposing factors for OC (Akpan and Morgan, 2002; Lu, 2021). Oral candidiasis is also a common infection in HIV-infected patients that is observed in more than 90% of these patients during HIV infection, especially in the early stages before highly active antiretroviral therapy (HAART) as well as in advanced stages of AIDS (Walmsley et al., 2001; Gabler et al., 2008). Oral thrush, erythematous candidiasis, angular cheilitis, and chronic hyperplastic candidiasis are the most prevalent clinical forms of oral candidiasis in people living with HIV/AIDS (PLWHA) (Shetti et al., 2011; Ribeiro Ribeiro et al., 2015).

Despite the fact that the incidence of opportunistic infections, including candidiasis, has dropped drastically after HAART (Ribeiro Ribeiro et al., 2015), the emergence of non-*albicans Candida* (NAC) species and the high incidence of antifungal-resistant *Candida* strains along with other opportunistic infections cause discomfort in PLWHA (Khedri et al., 2018). Oral candidiasis, often caused by *Candida albicans*, is one of the first clinical symptoms of human immunodeficiency virus, and the first AIDS patients were diagnosed due to OC symptoms (Shetti et al., 2011). OC diagnosis is essentially clinical, which is based on appearance and risk factors; isolation and identification of the causative yeast species is mostly done to confirm the diagnosis and select the appropriate treatment (Loo, 2009; Coronado-Castellote and Jiménez-Soriano, 2013). *Candida albicans* is the most commonly implicated species in OC (Taverne-Ghadwal et al., 2022), followed by *C. glabrata*, *C. tropicalis*, and *C. dubliniensis* that are considered important causative agents of OC (Patel et al., 2012; Patil et al., 2018).

In many developing countries such as Iran, OC treatment is performed without accurate diagnosis of *Candida* spp. and is only based on clinical signs (Taverne-Ghadwal et al., 2022). On the other hand, the prevalence of *Candida* spp. has shifted from *C. albicans* to NAC species during the last few decades (Terças et al., 2017; Song et al., 2020). Therefore, the epidemiology of OC in the present era of HAART is not well established (Patuwo et al., 2015). Thus, with the above outlook, the present study emphasizes on determining OC prevalence and changing *Candida* profiles to investigate the possible risk factors associated with OC in PLWHA following HAART treatment. Furthermore, we evaluated the relationship between OC and socio-demographic characteristics and clinical information of PLWHA in southwest Iran in an attempt to contribute to better management of infections in these patients.

## Materials and methods

### Study design and participants

This study is part of a cross-sectional research that was performed after obtaining written permission for sampling as well as ethical code (IR.AJUMS.MEDICINE.REC.1399.034) from the research committee of Ahvaz Jundishapur University of Medical Sciences in collaboration with Behavioral Diseases Counseling Center (BDCC). Under the auspices of Ahvaz Jundishapur University of Medical Sciences in southwest Iran, BDCC provides free services to PLWHA in the field of counseling, HIV/AIDS diagnosis, harm reduction, care and treatment. The sample size was calculated by assuming an expected prevalence (*p*) of 43.78% from the previous study of Aboualigalehdari et al. (2020) with 95% confidence using the following formula:

n=N×X⁢/⁢(X+N⁢-⁢1),

Where $X=Z_{\alpha /2}^{2}\times p\times \left(1-p\right)\,/\,d^{2}$, in which *z* = 1.96, 1 – *p* = 56.22% and *d* = 0.05. Therefore, the minimum sample size was computed as 267. Eventually, information from 276 individuals with HIV was included in the study. 450 out of 905 PLWHA covered by BDCC visited for their regular monthly health controls. All patients involved were informed about the aim of the study, and 276 active clients provided informed written consent to participate in the present research. All the participants were receiving HAART. The most common antiretroviral medications prescribed to BDCC patients were TRUVADA (emtricitabine and tenofovir disoproxil fumarate), followed by abacavir, lamivudine, zidovudine, and efavirenz. Unfortunately, there were no antifungal drugs in the center from 2 years before sampling of the present project. During oral sampling, the results were provided to physicians of BDCC so that they could prescribe fluconazole to the patients at their discretion. Oral samples were collected from October 22, 2020 up to April 21, 2021. Then, patients’ socio-demographic details, risk factors and clinical information were collected from BDCC information system. The presence of oral symptoms and the growth of *Candida* spp. on culture obtained from the oral cavity were considered indicative of OC (Osaigbovo et al., 2017).

### Inclusion and exclusion criteria

1. The participants whose HIV positivity had been confirmed by both enzyme-linked immunosorbent assay (ELISA) and Real-time PCR. Therefore, participants whose HIV infection was not confirmed by BDCC laboratory were excluded from the survey.
2. Individuals who presented informed consent to participate in the study; therefore, patients who were not willing to participate in the study with any reason(s) or those who had incomplete information were excluded from the sampling.
3. Patients who had one of the following symptoms: a feeling of change in the sense of taste or dry mouth, inflamed lesions in the oral mucosa and tongue with red scabs, a false white membrane in the oral cavity or creamy to white plaques.
4. Yeast growth with colony counts ≤ 10 and <10 after culture of oral swab on CHROMagar™ *Candida* medium were considered OC group and without OC group, respectively (Erköse and Erturan, 2007).
5. Molecular and macroscopic confirmation of *Candida* spp. samples isolated from the oral cavity of PLWHA.

### Sample collection and phenotypic identification

Oral samples taken from patients were immediately cultured on plates containing CHROMagar™ *Candida* medium (CHROMagar™, Pioneer, Paris, France) and incubated at 35°C for 48–72 h. The yeasts were then differentiated based on morphology and color of the colonies on a chromogenic medium, and different colonies were counted based on colony forming unit (CFU)/swab. Patients with ≥10 CFU/swab were defined as OC group, while participants with <10 CFU/swab and/or those showing growth of non-*candida* spp. such as bacterial isolates were considered the group without OC. Then the yeasts purified from the chromogenic medium were transferred to two series of microtubes containing sterile distilled water for long-term storage, stocked at room temperature and refrigerated. Isolates were also stored in tubes containing SDA medium (Sabouraud Dextrose Agar with chloramphenicol-Liofilchem, Italy) after incubation for 48 h at 35°C.

### DNA extraction and molecular identification

Genomic DNA of each strain was extracted by boiling. This method is an efficient, reproducible, fast and cheap approach not requiring chemical reagents or any purification procedures, which can simply yield a high-grade PCR product after at least 20 mins of boiling (Silva et al., 2012). The *ITS*1-5.8S rDNA-*ITS*2 region was amplified using 0.5 μl of the 10 μM universal fungal primers *ITS*1 (5’-TCC GTA GGT GAA CCT GCG G-3’) and *ITS*4 (5’-TCC TCC GCT TAT TGA TAT GC-3’) (Mirhendi et al., 2006; Mohammadi et al., 2013), 12.5 μl of 2 × Taq DNA master mix (Ampliqon), 3 μl of DNA template, and PCR- grade water were used to make a final reaction volume of 25 μl. The amplification program involved an initial step to denature the target DNA by heating at 95°C for 5 min. After the two intertwined strands of DNA were separated from each other, the single-stranded DNA template was replicated in 35 cycles of 35 s denaturation at 95°C, annealing for 30 s at 58°C and extension for 1 min at 72°C, followed by final extension at 72°C for 10 min. Subsequently, the products were digested with *Hpa*II restriction enzyme (*Msp*I; Thermo Fisher Scientific, Waltham, MA, United States). Other restriction enzymes have also been introduced by researchers to identify *Candida* species, but the patterns produced by *Hpa*II alone without the use of other enzymes lead to the differentiation of common *Candida* species (Deák et al., 2004; Pinto et al., 2004; Mirhendi et al., 2006). Digestion was performed according to manufacturer’s recommendations in a final reaction volume of 31 μl containing 10 μl of PCR product, 1 μl of enzyme, 2 μl of 10× buffers, 18 μl of DW. The mixture was incubated at 37°C for 1–16 h. Banding patterns obtained for each isolate were analyzed by comparing the profiles of *Candida* spp. in previous reports (Mohammadi et al., 2013). Next, for differentiation between *C. albicans* and *C. dubliniensis*, *ITS*1 and *ITS*2 region were amplified using two pairs of primers: *CAL*F (5′-TGG TAA GGC GGG ATC GCT T-3′), *CAL*R (5′-GGT CAA AGT TTG AAG ATA TAC-3′) for *C. albicans* and *CDU*F (5′-AAA CTT GTC ACG AGA TTA TTT TT-3′), *CDU*R (5′-AAA GTT TGA AGA ATA AAA TGG C-3′) for *C. dubliniensis*. The reaction contained 12.5 μL of 2X master mix (Amplicon), 0.5 μL of each primer (10 μM), 3 μl of DNA template and PCR-grade water to make a final reaction volume of 25 μl. Duplex PCR cycles included initial denaturation at 95°C for 5 min, 35 cycles of denaturation at 95°C for 30 s, annealing at 56°C for 45 s, extension at 72°C for 1 min, with a final extension step of 5 min at 72°C. The PCR product sizes of ∼100 bp and a ∼325 bp were identical to *C. albicans* and *C. dubliniensis*, respectively (Aboualigalehdari et al., 2020; Farahyar et al., 2020).

Also, a pair of primers (CR-f 5′-GCT ACC ACT TCA GAA TCA TCA TC-3′ and CR-r 5′-GCA CCT TCA GTC GTA GAG ACG-3′) was used for partial amplification of hyphal wall protein 1 (*HWP*1) gene. *HWP*1 is expressed in germ tubes and in true hyphae. This gene is a cell surface protein of *C. albicans* clade members, which cross links these germ tube-positive *Candida* spp. to epithelial cells (Romeo and Criseo, 2008). *HWP*1 is a target for rapid, high throughput and specific identification of each member of *C. albicans* clade using only a single pair of primers (Shokoohi et al., 2021). Each reaction was prepared in a final volume of 25 μl. PCR program involved the following steps: an initial denaturation at 95°C for 5 min, followed by 30 cycles at 94°C for 45 s, 58°C for 40 s, and 72°C for 55 s, final extension at 72°C for 10 min. The PCR product yielded a ∼941, ∼800, ∼740, and 569 bp fragments for *C. albicans*, *C. stellatoidea*, *C. africana*, and *C. dubliniensis*, respectively (Shokoohi et al., 2021).

### Statistical analysis

Statistical analysis was performed using the statistical software SPSS 22 (SPSS Inc., Chicago, IL, United States). The normality of continuous variables was examined using the Shapiro-Wilk’s W-test. Continuous variables are reported as mean ± SD. Categorical data are expressed as number (percentage). Two independent samples *t*-test or Man-Whitney test were used to compare the continuous variables between patients with OC and without OC. Furthermore, univariate and multiple binary logistic regression analyses were performed to calculate crude and adjusted odds ratios of the association between the explanatory variables and the OC status, with Odds ratios (OR), the respective 95% confidence intervals (CI) and *p*-values (likelihood ratio statistic). Variables associated with the outcome (with OC/without OC) with *P* < 0.20 in the univariate analysis were included in a multiple logistic regression model. Backward stepwise logistic regression modeling was then used to obtain a subset of factors associated with the OC status.

### Ethics statement

This study was performed after obtaining written permission for sampling and ethical code number (IR.AJUMS.MEDICINE.REC.1399.034) from research committee of Ahvaz Jundishapur University of Medical Sciences. All the participants signed informed consent forms to participate in the study.

## Results

**FIGURE 1:**
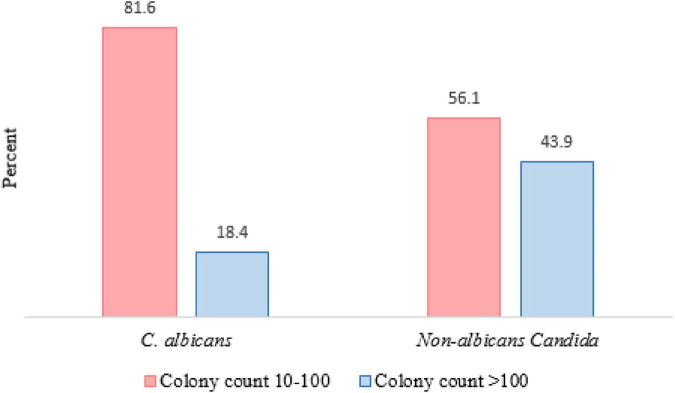
Comparison of colony counts between *Candida albicans* and NAC spp. among individuals with OC (*P* value = 0.008).

A total of 276 HIV/AIDS participants eligible for the study were included in the final statistical analysis. 113 patients had OC, which indicated a prevalence rate of 41%. 154 oral *Candida* spp. were isolated from 113 HIV/AIDS patients with OC. The distribution of species in Table 1 shows that *C. albicans* (64.6%) was the dominant species isolated in our study, followed by *C. glabrata* (26.5%) and *C. dubliniensis* (19.5%). *C. famata*, *C. africana*, and *C. stellatoidea* were first isolated from OC in PLWHA in southwest Iran. Furthermore, in 41 cases (36.3%) from 115 patients, mixed cultures of two to three species were observed. In general, the most dominant co-infection was *C. albicans* and/or *C. glabrata* in combination with non-*albicans* species (36/41, 87.80%). Additional details on co-infection are provided in Table 2. Colony count resulted in 3,665.13 ± 18,538 (mean ± SD) and 1.26 ± 1.96 colonies in OC and without OC groups, respectively (*p* < 0.0001). In Figure 1, the number of colonies was compared between *C. albicans* and NAC spp. among individuals with OC (*p* = 0.008).

**TABLE 1:** Distribution of *Candida* strains (*n* = 154) isolated from patients with OC (*n* = 113).

Species	*n*	Isolation rate by species, %	Isolation rate by patients, %	Colony color on CHROMagar*
*Candida albicans*	73	47.5	64.6	Green
*Candida glabrata*	30	19.5	26.5	Mauve
*Candida dubliniensis*	22	14.3	19.5	Green
*Candida tropicalis*	9	5.8	8.0	Metallic blue
*Candida famata*	7	4.5	6.2	Pink
*Candida kefyr*	4	2.6	3.5	Pink
*Candida krusei*	4	2.6	3.5	Pink and fuzzy
*Candida parapsilosis*	2	1.3	1.8	Pink
*Candida africana*	2	1.3	1.8	Green
*Candida stellatoidea*	1	0.6	0.9	Green

*The spectrum of green and pink colors vary in different species.

**TABLE 2:** Distribution of co-infection with different *Candida* species in 113 patients with OC.

Co-infection of species	Patients, *n* (%)
*Candida albicans* & *Candida glabrata*	11 (9.7)
*Candida albicans* & *Candida dubliniensis*	2 (1.8)
*Candida albicans* & *Candida africana*	2 (1.8)
*Candida albicans* & *Candida famata*	2 (1.8)
*Candida albicans* & *Candida stellatoidea*	1 (0.9)
*Candida albicans* & *Candida tropicalis*	1 (0.9)
*Candida albicans* & *Candida kefyr*	1 (0.9)
*Candida albicans* & *Candida krusei*	1 (0.9)
*Candida dubliniensis* & *Candida glabrata*	5 (4.4)
*Candida dubliniensis* & *Candida famata*	1 (0.9)
*Candida dubliniensis* & *Candida tropicalis*	1 (0.9)
*Candida glabrata* & *Candida tropicalis*	4 (3.5)
*Candida glabrata* & *Candida kefyr*	1 (0.9)
*Candida tropicalis* & *Candida parapsilosis*	1 (0.9)
*Candida famata* & *Candida kefyr*	1 (0.9)
*Candida famata* & *Candida krusei*	1 (0.9)
*Candida albicans* & *Candida famata* & *Candida krusei*	1 (0.9)
*Candida albicans* & *Candida glabrata* & *Candida dubliniensis*	1 (0.9)
*Candida albicans* & *Candida glabrata* & *Candida tropicalis*	1 (0.9)
*Candida albicans* & *Candida glabrata* & *Candida kefyr*	1 (0.9)
*Candida glabrata* & *Candida tropicalis* & *Candida parapsilosis*	1 (0.9)
Total co-infection	41 (36.3)

There were 168 (60.9%) male and 108 (39.1%) female patients with female to male ratio of 1:1.55. The average age was 38.93 years. The mean body mass index (BMI) and body weight was 25.29 kg/m^2^ and 71.54 **±** 9.5 kg, respectively. Univariate logistic regression analyses indicated a significant association between BMI and the occurrence of OC (OR = 0.0917; CI = 0.87–0.96; *p* = 0.001). Also, male patients (OR = 1.960; CI = 1.02–2.79; *p* = 0.04), individuals with a history of incarceration (OR = 1.886; CI = 1.14–3.09; *p* = 0.012), condom use (OR = 1.556; CI = 0.82–2.93; *p* = 0.173), sex with multiple partner(s) (OR = 1.518; CI = 0.90–2.50; *p* = 0.118), addiction (OR = 1.930; CI = 1.18–3.14; *p* = 0.008), intravenous drug abusers (IVDA) (OR = 2.330; CI = 1.42–3.82; *p* = 0.001), CD4 count ≤ 200 cells/mm^3^ (OR = 4.736; CI = 2.02–11.07; *p* < 0.001), WHO clinical stage IV (OR = 4.324; CI = 1.31–14.27; *p* = 0.054), treatment history of HCV infection (OR = 2.092; CI = 1.23–3.54; *p* = 0.006), PCP (OR = 1.530; CI = 0.94–2.49; *p* = 0.087) and other infections (OR = 1.980; CI = 1.21–3.22; *p* = 0.006) were significantly more likely to have OC. A significant association was found between other factors and OC in PLWHA (Table 3). According to the results of multiple logistic regression analyses, BMI and CD4 count ≤ 200 cells/mm^3^ were the only predictors of OC in the final model (Table 4).

**TABLE 3:** The important risk factors for oral Candidiasis (univariate logistic regression).

Variable	Without OC (*N* = 163)	With OC (*N* = 113)	OR (95% CI)	*P*-value (<0.20)
**Socio-demographic characteristics**				
**Sex**				0.04
Male	91 (55.8)	77 (68.1)	1.960 (1.02–2.79)	
Female	72 (44.2)	36 (31.9)	Ref	
**Nationality**				0.703
Iranian	159 (100)	111 (100)	1.390 (0.25–7.75)	
Non-Iranian	4 (2.5)	2 (1.8)	Ref	
**Age** (years)*	38.53 ± 10.65	39.51 ± 10.35	1.009 (0.98–1.03)	0.431
**Weight** (kg)*	73.69 ± 16.58	68.44 ± 17.98	0.982 (0.96–0.99)	0.015
**BMI** (kg/m^2^)*	26.23 ± 5.08	23.93 ± 5.35	0.0917 (0.87–0.96)	0.001
**Level of education**				0.071
Illiterate	13 (8.0)	15 (13.3)	Ref	
Primary and middle school	95 (58.3)	73 (64.6)	0.666 (0.29–1.48)	
Secondary school and higher education	55 (33.7)	25 (22.1)	0.394 (0.16–0.95)	
**History of incarceration**				0.012
Neg	111 (68.1)	60 (53.1)	Ref	
Pos	52 (31.9)	53 (46.9)	1.886 (1.14–3.09)	
**Marital status**				0.026
Unmarried	57 (35.2)	55 (48.71)	Ref	
Married	105 (64.8)	58 (51.29)	0.572 (0.35–0.93)	
**Condom use**				0.173
Neg	140 (58.9)	90 (79.6)	Ref	
Pos	23 (14.1)	23 (20.4)	1.556 (0.82–2.93)	
**Sex with multiple partner(s)**				0.118
Neg	121 (74.2)	74 (65.5)	Ref	
Pos	42 (28.5)	39 (34.5)	1.518 (0.90–2.50)	
**Heterosexual**				0.793
Neg	113 (69.3)	80 (70.8)	Ref	
Pos	50 (30.7)	33 (29.2)	0.932 (0.55–1.57)	
**Homosexual**				0.599
Neg	159 (97.5)	109 (96.5)	Ref	
Pos	4 (2.5)	4 (3.5)	1.459 (0.53–5.95)	
**Addiction**				0.008
Neg	93 (57.1)	46 (40.7)	Ref	
Pos	70 (42.9)	67 (59.3)	1.930 (1.18–3.14)	
**IVDA**				0.001
Neg	111 (68.1)	54 (47.8)	Ref	
Pos	52 (31.9)	59 (52.2)	2.330 (1.42–3.82)	
**Needle sharing**				0.026
No	115 (70.6)	65 (57.5)	Ref	
Yes	48 (29.4)	48 (42.5)	1.769 (1.07–2.92)	
**Clinical tests**				
**CD4 count** (cells/mm)^3^				<0.0001
≤200	8 (4.9)	22 (19.6)	4.736 (2.02–11.07)	
>200	155 (95.1)	90 (80.4)	Ref	
**WHO clinical stage**				0.054
Stage 1	128 (78.5)	74 (65.5)	Ref	
Stage 2	19 (11.7)	17 (15.0)	1.548 (0.75–3.16)	
Stage 3	12 (7.4)	12 (10.6)	1.73 (0.73–4.04)	
Stage 4	4 (2.5)	10 (8.8)	4.324 (1.31–14.27)	
**Chest X-ray**				0.023
Abnormal	40 (29.9)	44 (44.4)	Ref	
Normal	94 (70.1)	55 (55.6)	0.532 (0.30–0.91)	
**AST** (U/L)*	31.54 ± 20.15	37.13 ± 41.89	1.000 (0.99–1.01)	0.175
**ALT** (U/L)*	35.72 ± 41.13	33.21 ± 37.56	0.998 (0.99–1.00)	0.609
**ALP** (U/L)*	321.85 ± 133.15	356.02 ± 210.73	1.000 (1.00–1.01)	0.113
**RBC** (cells/mm^3^)*	4,715,341.62 ± 6,58,598.92	4,581,851.85 ± 623,532.51	1.000 (0.99–1.00)	0.098
**Treatment history**				
**HCV infection**				0.006
Neg	126 (77.3)	70 (61.9)	Ref	
Pos	37 (22.7)	43 (38.1)	2.092 (1.23–3.54)	
**TB**				0.351
Neg	133 (81.6)	87 (77.0)	Ref	
Pos	30 (18.4)	26 (23.0)	1.32 (0.73–2.39)	
**HBV infection**				0.841
Neg	158 (96.9)	110 (97.3)	Ref	
Pos	5 (3.1)	3 (2.7)	0.862 (0.20–3.68)	
**PCP**				0.087
Neg	102 (62.6)	59 (52.2)	Ref	
Pos	61 (37.4)	54 (47.8)	1.530 (0.94–2.49)	
**Other infections** **				0.006
Neg	101 (61.9)	51 (45.1)	Ref	
Pos	62 (38.1)	62 (54.9)	1.980 (1.21–3.22)	

*Values presented as Mean ± SD and others stated Number (%). **The infections included Herpes zoster, Cytomegalovirus, Kaposi sarcoma, progressive multifocal leukoencephalopathy, chronic diarrhea, severe bacterial infection, cerebral toxoplasmosis, fungal infections (except PCP and OC). BMI, body mass index; IVDA, intravenous drug abuser; AST, aspartate transaminase; ALT, alanine aminotransferase; ALP, alkaline phosphatase; RBC, red blood cell; HCV, hepatitis C virus; TB, tuberculosis; HBV, hepatitis B virus; PCP, pneumocystis pneumonia; Neg, negative; Pos, positive.

**TABLE 4:** The important risk factors for oral Candidiasis (multiple logistic regression).

Variable	OR (95% CI)	*p*-value (<0.05)
BMI	0.947 (0.89–0.99)	0.045
Incarceration history		0.090
Neg	Ref	
Pos	1.59 (0.93–2.72)	
CD4 count		0.002
≤200	4.365 (1.73–10.98)	
>200	Ref	
ALP	1.001 (1.00–1.00)	0.085

BMI, body mass index; ALP, alkaline phosphatase.

## Discussion

Oral candidiasis is an immunological state marker of HIV-positive patients and therefore represents a clinical predictor of HIV infection progression (Suryana et al., 2020). However, limited investigations in Iran have examined the prevalence of *Candida* species and associated factors in the oral cavity of HIV infected individuals (Ayatollahi Mousavi et al., 2012; Sharifzadeh et al., 2013; Khedri et al., 2018; Aboualigalehdari et al., 2020). In this survey, OC affected 41% of patients. Similar findings have been found in other recent studies in Iran (Khedri et al., 2018; Aboualigalehdari et al., 2020) and in other countries (Goulart et al., 2018; Ambe et al., 2020; Suryana et al., 2020), which showed that the rate of OC in PLWHA was 40–50%. Compared to previous studies conducted in Iran by Badiee et al. (2010) (71.5%), Ayatollahi Mousavi et al. (2012) (69%), and Katiraee et al. (2010) (59%), the prevalence rate has decreased. HAART has been proven to restore/maintain immune function and significantly reduce the risk of mortality (Dore and Cooper, 2006). There has been a marked decrease in HIV-related opportunistic infections, including OPC from 1990s when HAART was introduced (Patil et al., 2018). In our country, the declining prevalence of HIV reflects the introduction and widespread use of HAART, improvement of immunological response in PLWHA, subsequent viral load suppression and decreased frequency of opportunistic infections such as OC. However, the epidemiology of OC in ART era in Iran demonstrated that even with therapeutic advances for HIV treatment, the prevalence of oral candidiasis still remains a challenge and the patients continue to suffer significant morbidity of OC.

*Candida albicans* was the most frequent *Candida* species isolated (64.6%), which occurred more due to its pathogenic mechanism factors, followed by NAC species (35.4%). In two studies among Iranian HIV positive populations, the frequency of *C. albicans* was 60 and 69.3%, respectively, which are in accordance with our analysis (Katiraee et al., 2015; Aboualigalehdari et al., 2020). A point to consider in the analyses on OC group was the lower colony count of *C. albicans* compared to NAC spp. Thus, *C. albicans* could cause infection with low colony counts, which confirms the high expression of virulence factors in *C. albicans*. In a review study conducted in 2018, Patil et al. (2018) reported a great variety of NAC among HIV infected patients probably due to the differences in sampling, diagnosis method, climate conditions, ethnicity and other underlying factors (Abharian et al., 2020; Aboualigalehdari et al., 2020). The presence of *C. glabrata* as the second most common and dominant NAC species in many studies can be regarded as a parallel finding in these researches (Junqueira et al., 2012; Patel et al., 2012; Katiraee et al., 2015; Goulart et al., 2018; Ambe et al., 2020). Previous studies have indicated that species such as *C. glabrata* and *C. krusei* are less susceptible or resistant to some antifungal agents (Arastehfar et al., 2019; Rajadurai et al., 2021). As a co-infecting agent or the sole detectable species from oral lesions associated OC infections in HIV-positive patients, these species tend to be more severe and more difficult to treat than infections due solely to *C. albicans* (Junqueira et al., 2012; Aboualigalehdari et al., 2020). Therefore, the presence of co-infections or shift toward NAC infections can be clinically challenging.

We found that BMI was a statistically significant risk factor for OC among PLWHA (OR = 0.0917; CI = 0.87–0.96; *p* = 0.001). Previous investigations have shown that low BMI (<18.5 kg/m^2^) was associated with treatment failure (failure to increase CD4 count or to suppress viral load) and increased mortality in PLWHA (Liu et al., 2011; Evans et al., 2012). In this way, Jiang et al. (2019) found that cumulative mortality was lower in both overweight BMI and normal BMI than in the low BMI, with an adjusted hazard ratio (AHR) of 0.3 (CI = 0.1–0.6; *p* = 0.002) and 0.5 (CI = 0.4–0.7; *p* < 0.001), respectively. The results of relationship between BMI and the risk of OC among PLWHA in this analysis supports the findings of another study in which Evans et al. revealed that low BMI was associated with OC at HAART initiation. It also demonstrated that low-BMI patients with OC at initiation of HAART have poor treatment outcomes compared to those without OC having normal BMI (OR = 0.92; CI = 0.80–1.06) (Evans et al., 2012). In addition, a survey conducted in Tanzania on oral manifestations among 187 persons with HIV infection showed a significant relationship between low BMI and presence of OC (*P* < 0.01; Fabian et al., 2009). In this research, 14.3% of patients had low BMI in OC group, while this rate was only 6.3% in patients without OC. BMI, and especially low BMI, seems to be particularly important in the management of HIV infection. In general, the mean BMI of the group without OC is higher than the OC group and is classified in overweight BMI. Therefore, it seems that participants with low and even normal BMI have a higher chance of developing OC than patients with overweight BMI. This could possibly be explained by incidence of OC in advanced stage of HIV infected people having malnutrition. Meanwhile, it could also indicate that oral discomfort of OC leads to impaired feeding, which in turn causes loss of body weight (Hilton et al., 2004; Fabian et al., 2009).

Numerous publications documented a relation between OC with decreased CD4 counts (Li et al., 2013; Berberi et al., 2015; Takahashi et al., 2015; Goulart et al., 2018; Ambe et al., 2020; Suryana et al., 2020). In the present study, a significant relationship was observed between OC and CD4^+^ count ≤ 200 cells/mm^3^ through multiple logistic regression analysis (OR = 4.365; CI = 1.73–10.98; *P* = 0.002). Therefore, CD4 count ≤ 200 cells/mm^3^ had approximately 4.5 times higher risk of OC development than CD4 count ≤200 cells/mm^3^. Low CD4 counts in HIV patients indicate a low immune status or immunosuppression, and our findings, consistent with other surveys, emphasize that OC can be a clinical indicator of HIV infection in individuals who do not know their HIV status or those experiencing failed treatment.

According to WHO, HIV clinical stage demonstrates patients’ degree of immunosuppression and an increasing susceptibility to opportunistic infections, and the severity of HIV progression may be responsible for the increase in OC by *Candida* spp. Our results showed that OC odds increased >4-fold for patients with clinical WHO stage IV compared to those with WHO stage I (OR = 4.324; CI = 1.31–14.27; *p* = 0.054). Nanteza et al. (2014) also revealed that individuals under WHO stage III are almost four times more likely to develop oral candidiasis (OR = 3.803; CI = 1.182–12.240; *p* = 0.025). In the study of Suryana et al. (2020), WHO clinical stage II-IV had an association with OC compared to WHO stage I (OR = 3.58; CI = 2.39–5.37; *P* = 0.000).

Despite our prospects, no statistical difference was found in age between PLWHA with and without OC (OR = 1.009; CI = 0.98–1.03; *P* = 0.431), which could be explained by the fact that a majority of the study participants were in the age group of 21–40 years (52.2). This result is in line with studies carried out in Iran (Khedri et al., 2018) and Cameroon (Ambe et al., 2020), but it is contrary to other studies indicating that OC is age dependent (Aboualigalehdari et al., 2020; Suryana et al., 2020).

On the other hand, the odds of OC were increased 2-fold for males compared with females (OR = 1.960; CI = 1.02–2.79; *P* = 0.04). Similar to our results, Suryana et al. (2020) demonstrated that OC cases in males were significantly higher than in females (OR = 1.88; CI = 1.26–2.80; *p* = 0.002). This may be related to the potential risk factors of acquiring HIV transmission such as addiction, poor oral hygiene and high-risk sex, which are more common in males than in females of the Iranian population (Ghamari et al., 2011; Asgari et al., 2015; Sajadipour et al., 2022).

In this study, addiction, IVDA, and needle sharing significantly increased the risk of OC occurrence by nearly 2-fold (Table 3). According to current claims, addiction causes local and systemic ineffective immune system and provides conditions for increasing pathogen acquisition and development of OC (Specter, 1994; Friedman et al., 2006; Abharian et al., 2020).

The significant association between OC and sexual behaviors [such as condom use, sex with multiple partner(s), and homosexuality] in this analysis was probably due to the fact that these individuals may also spread OC exogenously.

Univariate analysis revealed that increased alkaline phosphatase (ALP) level was the risk factor of developing OC in PLWHA. This increase was found to be statistically significant (*p* = 0.113). ALP is an enzyme found throughout the body in healthy individuals, but serum ALP is primarily derived from liver and bones (Sharma et al., 2014). Several factors cause increased ALP levels. Tenofovir, inhaled opium, and cigarette smoking cause elevated ALP levels (Abongwa et al., 2022; Bijani et al., 2022). Tenofovir is used to prevent and treat HIV/AIDS as well as chronic hepatitis B. Fux et al. (2008) documented a significant association between tenofovir use and elevated levels of bone isoenzyme of ALP (*p* ≤ 0.003) in HIV patients. These finding raises concerns about rising ALP levels in HIV patients and drug addicts. In this regard, it is suggested that ALP should be measured regularly in the mentioned groups, especially during OC.

Our analysis revealed that participants with normal chest X-ray (CXR) were approximately 50% less likely to develop OC than those with abnormal chest graph (OR = 0.532; CI = 0.30–0.91; *p* = 0.023). Opportunistic infections including Pneumocystis pneumonia (PCP), tuberculosis (TB) or neoplasm have a characteristic radiographic presentation (Padyana et al., 2012). As a result, CXR combined with other clinical information such as OC, which is an indicator of immunosuppression, can limit diagnostic possibilities and suggest a diagnosis.

The risk of opportunistic lung infections increases with impaired immune systems. Based on the results of the present study, the chance of developing OC in PLWHA with PCP is approximately 1.5 times higher (OR = 1.530; CI = 0.94–2.49; *p* = 0.087). PCP is one of the most frequent opportunistic infection in PLWHA; however, because of its non-specific signs and symptoms, a high clinical suspicion is the single most important diagnostic tool for early diagnosis of PCP (Tasaka, 2015; Ko et al., 2020). We recommend that HIV positive patient with OC should be checked for PCP and receive prophylaxis for it.

In our study, HCV therapy led to 2-fold higher occurrence of OC (OR = 2.092; CI = 1.23–3.54; *p* = 0.006). All patients with co-infection of HCV and HIV were treated with interferon (IFN) therapy. Previous surveys have demonstrated that salivary flow is decreased throughout the course of IFN treatment in all individuals (Nagao et al., 2012). Decreasing saliva production can increase the risk of diseases such as OC (Suryana et al., 2020). Yakoob et al. (2003) found that *Candida* esophagitis in a patient with chronic HCV infection suggests immune suppression because of HCV, which leads to oral opportunistic infections.

The strengths of this study include the evaluation of detailed HIV information and attempting to accurately discriminate between different species. Some species such as *C. africana*, *C. stellatoidea*, and more importantly, *C. dubliniensis* are frequently identified species in PLWHA. The limitations of this study were that the latest viral load could not be accessed and that not all opportunistic diseases were analyzed separately. This study has a cross-sectional design, so another limitation was the lack of true understanding of causal pathways; therefore, we can only comment on risk factors as being associated with OC.

## Conclusion

Taken together, our findings pointed out that OC still remains a significant opportunistic infection in post-HAART era among PLWHA, which is related to host factors. Ten *Candida* spp. were identified, but *C. albicans* (64.6%) has been the most predominant species. CD4 count ≤ 200 cells/mm^3^ and BMI were independent risk factors for OC. In the present study, an attempt was made to propose the most effective monitoring of patients by providing the analyzed data.

## Data availability statement

The raw data supporting the conclusions of this article will be made available by the authors, without undue reservation.

## Ethics statement

The studies involving human participants were reviewed and approved by ethical approval: IR.AJUMS.REC.1399.034. Written informed consent to participate in this study was provided by the participants’ legal guardian/next of kin.

## Author contributions

MF was involved in the study design and interpretation of the study’s data and the final editing of the manuscript. ME contributed to all the steps of experimental work, collection, and preparation of clinical samples, data analysis, and preparation of the manuscript draft. AZ contributed interpretation of the data. EM contributed study sampling design and statistical calculations. MH contributed in molecular analysis. All authors contributed to the article and approved the submitted version.

## Abbreviations

OC: oral candidiasis
PLWHA: people living with HIV/AIDS
HAART: highly active antiretroviral therapy
NAC: non-*albicans Candida;*
BDCC: Behavioral Diseases Counseling Center
OR: odds ratios
CI: 95% confidence intervals
BMI: body mass index.
